# A Gender Perspective on Coloproctological Diseases: A Narrative Review on Female Disorders

**DOI:** 10.3390/jcm13206136

**Published:** 2024-10-15

**Authors:** Paola De Nardi, Greta Giacomel, Simone Orlandi, Giulia Poli, Mauro Pozzo, Marcella Rinaldi, Antonella Veglia, Renato Pietroletti

**Affiliations:** 1 Gastrointestinal Surgery, IRCCS San Raffaele Scientific Institute, 20132 Milan, Italy; 2 General Surgery, San Vito al Tagliamento Hospital, 33078 San Vito al Tagliamento, Italy; giacomelgreta1983@gmail.com (G.G.); giulia.poli@gmail.com (G.P.); 3 Gastroenterology and Endoscopy, Sacro Cuore Don Calabria Hospital, 37024 Negrar, Italy; simone.orlandi@sacrocuore.it; 4 General Surgery, Coloproctology Unit, Hospital of Biella-Ponderano, 13875 Ponderano, Italy; doc69@virgilio.it; 5 Department of Emergency and Transplant, Policlinico of Bari, 70124 Bari, Italy; marcella.rinaldi@uniba.it; 6 Surgery Unit, Cardarelli Hospital, 86100 Campobasso, Italy; antonella.veglia@asrem.org; 7 Surgical Coloproctology, Hospital Val Vibrata Sant’Omero, 64027 Teramo, Italy; renato.pietroletti@univaq.it; 8 Department of Applied Clinical and Biotechnological Sciences, University of L’Aquila, 67100 L’Aquila, Italy

**Keywords:** female, gender, colorectal disease, proctological diseases, pregnancy

## Abstract

Coloproctological diseases, including both benign and malignant conditions, are among the most common diagnoses in clinical practice. Several disorders affect both men and women, while others are unique to women, or women are at a greater risk of developing them. This is due to anatomical, biological, and social conditions and also due to females’ exclusive capabilities of reproduction and pregnancy. In this context, the same proctological disease could differ between men and women, who can experience different perceptions of health and sickness. There is a raised awareness about the impact of different diseases in women and a growing need for a personalized approach to women’s health. In this review, we aim to summarize the specific features of the main coloproctological diseases, specifically in the female population. This includes common complaints during pregnancy, conditions linked to vaginal delivery, functional consequences after colorectal resections, and conditions presenting a gender disposition.

## 1. Introduction

Coloproctology includes a spectrum of both benign and malignant conditions affecting the colon, rectum, and anus. Such diseases affect a large part of the world’s population and commonly present in the primary care practice. Both organic and functional diseases are among the most prevalently diagnosed disorders with an impact on health care resources and socio-economic costs.

Several diseases are particular to women due to anatomical differences between sexes. Female specific conditions, such as intestinal endometriosis or recto-vaginal fistula, could lead to long-standing complaints and can severely affect social relationships and quality of life.

Most conditions, though affecting both males and females, have different incidences, symptoms, or severity or present different responses to therapy depending on sex [1]. Nevertheless, including for prevalent illnesses such as colorectal cancer, gender differences are poorly studied, and guidelines lack sex-specific recommendations. Traditionally, medical research and clinical trials have been conducted in men, with the assumption that men and women are biologically identical. As a result, the genetic and gender peculiarities of women are often underestimated, and medicine is less evidence-based for women than for men [2]. Furthermore, common coloproctological affections occurring during pregnancy pose specific problems and might need to be treated differently. The risk of the disease should be balanced with the risk for the mother and the fetus; management should be integrated with the obstetrician and the patient herself, and undertaken in a multidisciplinary manner, [3] particularly if a surgical treatment is planned.

In 2019, in Italy, the Minister of Health formally approved the “Plan for the application and diffusion of gender medicine” in the national territory. Although interest in gender medicine is spreading throughout the world, with the approval of this law, Italy was the first Country in Europe to formalize the inclusion of the concept of “gender” in medicine.

This is the first review addressing coloproctological diseases in women, including diseases affecting the colon, rectum, or anus exclusively in women and common diseases occurring during pregnancy or childbirth or arising afterwards. With this review, we would contribute to the development of gender medicine in the field of coloproctology and draw the attention of healthcare professionals facing these diseases.

## 2. Materials and Methods

We searched PubMed for papers published in English, with all study designs, using “sex” or “gender” and the name of the disease of interest or the wide terms “disease” or “condition”. No time restrictions were applied. Only peer-reviewed publications were included. The following conditions were considered: rectal endometriosis, obstetric anal sphincter lesions, fertility and pregnancy in patients with IBD, hemorroids during pregnancy and delivery, urinary and sexual dysfunctions after rectal cancer surgery, and rectovaginal fistula. For each condition epidemiology, clinical presentation and management are reported.

## 3. Results

### 3.1. Rectal Endometriosis

#### 3.1.1. Definition and Prevalence

Endometriosis is an estrogen-dependent, chronic gynecological disorder associated with the presence of uterine endometrial tissue outside of the normal location—mainly on the pelvic peritoneum but also on the ovaries and in the rectovaginal septum. The prevalence of pelvic endometriosis is about 6–10% in the general female population, reaching 50% in women with subfertility. The disorder is most commonly diagnosed in women of reproductive age, although a diagnostic delay is typical (mean 11.7 years in the USA) due to the variability in symptoms and signs and diagnostic confusion [4].

Endometriosis can take one of three forms, depending on the clinical presentation and management: peritoneal endometriosis, ovarian endometriosis, or deep infiltrative endometriosis (DIE). DIE is the most aggressive form, defined as the presence of endometriotic implants that are over 5 mm in depth under the peritoneal surface, which affects 20% of women who suffer from endometriosis [5].

The most common sites for deep endometriosis include the uterosacral ligaments, the Douglas pouch, the rectum, the rectovaginal septum, the bladder, and the ureters.

Bowel endometriosis can be found in 12% of patients, with prevalence rising to 37% for patients presenting to referral centers. The main locations of intestinal endometriosis are the rectum and the recto-sigmoid junction (65.7%), the sigmoid (7.4%), and the appendix (6.4%).

#### 3.1.2. Symptoms

While DIE has a strong association with pelvic pain symptoms, namely dysmenorrhea, dyspareunia, and chronic pelvic pain, rectal endometriosis is specifically associated with dyschezia (painful defecation) and bright red blood per rectum during a period [6]. Chronic pain may be accompanied by other symptoms that include low back pain, nausea, distention, early satiety, and/or constipation. Melena, obstructive symptoms, or even intussusceptions are related to endometriotic implants above the rectum [7].

#### 3.1.3. Diagnosis

The pre-operative workup commonly includes transvaginal ultrasound (TVU) and magnetic resonance imaging (MRI). In trained hands, TVU serves as an effective initial imaging test for diagnosing posterior compartment pelvic endometriosis. The lesions observed can range from microscopic foci to larger nodules, frequently surrounded by smooth muscle hyperplasia and fibrosis. Implants of endometriosis typically involve the bowel wall from the serosa inwards, often sparing the mucosa. However, TVU offers a more restricted field of view compared to MRI, which can limit its ability to detect the full extent of the disease. For this reason, work-up is completed by a second-line tool such as the MRI, which allows an accurate disease mapping with complete abdominal-pelvic survey, demonstrating multifocal involvement [8].

According to the specific clinical scenario, different additional diagnostic tools may be required, such as transrectal ultrasound (e.g., low rectal lesions) or colonoscopy (e.g., rectal bleeding, altered bowel habits, obstructive symptoms).

#### 3.1.4. Treatment

Once the diagnosis has been confirmed, initial management is directed towards pain control and hormone therapy; this treatment can be useful in reducing symptoms even if it does not cure the disease.

In the context of a multidisciplinary approach, colorectal surgery becomes necessary for symptom relief, complete eradication of disease, and fertility improvement. Other indications for surgery include exclusion of malignancy and intussusception/obstruction [9].

Minimally invasive techniques have been demonstrated to be useful in reducing blood loss, decreasing pain scores, improving cosmetic results, and allowing for quicker recoveries. For these reasons, a laparoscopic approach is considered the gold standard. Recently, robotic platforms have also been gaining popularity, offering improved depth perception with 3D imaging, improved range of motion with wrist articulation, and motion scaling to minimize tremors and improve ergonomics [10].

Shave excision, discoid excision, and segmental bowel resection, with or without a nerve-sparing technique, are all feasible management options for surgical treatment of bowel endometriosis.

Rectal or bowel shaving is the most conservative approach and can be used for superficial and smaller lesions, typically < 3 cm. The goal is to remove any nodules and associated fibrosis, ideally without entering the bowel lumen. Compared to the other approaches, bowel shaving has the lowest postoperative complication rate (2.4%), recurrence rates ranging around 6.5%, and less chance of long–term postoperative bladder and bowel dysfunctions [11]. Disc excision consists of full-thickness resection of the lesion with primary closure of the defect using a 29, 31, or 33 mm transanal circular stapler or by direct suture. This approach is well suited for full-thickness lesions but should be avoided in lesions bigger than 3 cm to prevent stenosis or stricture. Disc excision is a safe approach, with a mean recurrence rate of 2.2%; the most common complication is rectal bleeding, which is usually self-limiting, but can be effectively managed by rectal endoscopy if needed [12].

Laparoscopic resection of the affected bowel with primary anastomosis is ideal for full-thickness, large, multifocal, circumferential, or obstructive lesions, and it is burdened by a postoperative complication rate of 10% to 15% [13]. After bowel resection, endometriosis recurrence is low (1–8%); however, segmental resection also carries the risk of bowel denervation, loss of compliance, or hypersensitivity [9,14], resulting in anal incontinence, major dyschezia, and fecal urgency [8,13], thus severely affecting the quality of life of women. Although some symptoms persist after resection, and some patients complain of a LARS-like syndrome, the quality of life nonetheless significantly improves after surgery [15].

### 3.2. Obstetric Anal Sphincter Lesions

#### 3.2.1. Incidence

Perineal injury during childbirth is a common event with a remarkable morbidity. Pelvic diaphragm lesions occur in 41% of vaginal deliveries and about 30% of para 1+ suffer or have suffered from urinary incontinence. “Occult” lesions of the sphincter are detected in 20–44% of cases, with fecal incontinence occurring in half of the cases. Midwifes and gynecologists may miss up to 87% and 28% of external sphincter lesions, respectively. Posterior perineal trauma, encompassing any injury to the posterior vaginal wall, perineal muscles, or anal sphincters, can result in complex damage [16,17]. Injuries to the perineum and sphincters during labor and vaginal delivery are typically caused by two primary mechanisms: tears in the striated perineal muscles and neurogenic damage to the pelvic floor nerve supply [18]. The majority of neuromuscular injuries tend to resolve on their own within the first year following delivery [19].

#### 3.2.2. Definition

According to the Royal College of Obstetrics and Gynecologists (RCOG) Classification, first-degree lacerations involve only superficial perineal tissues. Second-degree lacerations involve superficial perineal tissues and muscles of the central perineal body, without the involvement of the anal sphincters and the rectal mucosa. OASIs, the acronym for obstetric anal sphincter injuries, is used to describe third- and fourth-degree tears. Third-degree lacerations are classified based on the involvement of the anal sphincter muscles (3a less than 50% of external anal sphincter, 3b more than 50% of external anal sphincter, 3c external and internal anal sphincter involvement). Fourth-degree tears involves anal sphincters and rectal mucosa [20].

#### 3.2.3. Assessment

The incidence of OASIs ranges between 0.5% and 17% with an incidence of 5.7% in primiparae compared with 1.5% in multiparae [21]. In the last years, the reported rate has been increasing; this may be due to the systematic digital rectal examinations routinely performed after vaginal birth by well-trained medical-obstetrical staff who are able to detect and assess perineal, vaginal, and rectal injuries. This emphasizes the importance of conducting a thorough physical examination immediately after delivery. Accurate clinical evaluation, including a perineal inspection and digital rectal examination, remains the most reliable method for early detection of OASIs [22]. Endoanal ultrasound (EAUS) is considered the gold standard for identifying both external and internal anal sphincter injuries, as well as for assessing the location and extent of the damage [23]. The use of endoanal and external MRI to detect anal sphincter injuries has also been studied. Compared to EAUS, endoanal MRI shows lower sensitivity in diagnosing internal anal sphincter defects, though both methods are comparable in evaluating external anal sphincter injuries [18,24]. A study comparing external phased-array MRI with EAUS found that the former is more sensitive in detecting external anal sphincter defects and is a viable tool for diagnosing OASIs [25]. Among recent advancements, impedance spectroscopy has emerged as a promising technique for early OASI diagnosis, offering ease of use even by untrained personnel [26].

#### 3.2.4. Risk Factors

Many risk factors can be identified for OASIs, including ones pertaining to maternal, fetal, and childbirth reasons. The main maternal risk factors are a history of OASIs, Asiatic ethnicity, perianal lesions (perianal fissures, anorectal, or recto-vaginal fistula) and genital mutilations. Intrapartum risk factors include precipitate or a faster-than-expected second stage, prolonged labor (more than one hour), instrumental delivery (forceps, vacuum), epidural anesthesia, midline episiotomy or inadequately angled medio-lateral, which may end up like a mid-line episiotomy, and inappropriate maternal position such as sustained lithotomy position. Concerning the fetal risk factors, this includes fetal weight greater than 4 kg, posterior cephalic positions, birth beyond term, fetal suffering, and shoulder dystocia [27].

#### 3.2.5. Prevention

To decrease the incidence and severity of trauma, as well as to enhance both short- and long-term maternal outcomes related to perineal injury and recovery, all pregnant women should be involved in a prevention program. Three phases of prevention could be considered: pre-partum, intra-partum, and post-partum. In the pre-partum, all patients are assessed to detect possible disorders of the pelvic floor or other risk factors for the occurrence of perineal trauma. Patients considered to have no risk are invited to attend meetings dealing with the appropriate nutrition and style for everyday activities (urination, defecation, movements) and a prenatal course to learn the correct use of perineal muscles. Conversely, pelvic floor muscle training (PFMT) should be recommended during pregnancy and after births in patients with risk factors, usually involving one-third of women [28]. Antenatal digital perineal massage reduced by 9% the rate of perineal trauma requiring suturing and by 16% the likelihood of episiotomies and the documentation of persistent perineal pain. Generally, it is well accepted by women [29]. Intrapartum prevention strategies involve non-surgical techniques using hand maneuvers designed to reduce the diameter of the emerging head and slow its descent, allowing the perineal tissues to stretch gradually. These include “hands off” or “hands on” techniques, which reduce the rate of episiotomy, as well as the use of warm compresses and perineal massage. Perineal massage involves inserting two fingers into the vagina and performing firm, slow, even strokes to help stretch the perineum, which is believed to improve circulation and promote tissue relaxation and stretching [30]. Additionally, pushing techniques—whether coached or spontaneous—maternal birthing positions, and water birth may be considered for their potential impact on reducing perineal trauma.

As far as episiotomy is concerned, there are nowadays different attitudes: if in the past there was a liberal application of such a procedure, now it is indicated only in patients with a high likelihood of severe lacerations, as recommended by the RCOG. In the case of instrumental deliveries, or when needed, a mediolateral episiotomy angled 45–60 degrees from the midline should be considered, as it is linked to a lower risk of sphincter injuries compared to spontaneous lacerations [16,31,32]. Forced supine or semi-supine position for birth is discouraged; conversely, women are advised to choose a position based on their comfort. Postpartum prevention is indicated in all patients with a point equal to or greater than four by the perineal card: no therapy (from 0 to 3); group therapy (from 4 to 8); individual therapy (points more than 8).

#### 3.2.6. Treatment

When OASI is identified immediately after vaginal delivery, surgical repair is performed as soon as possible, known as primary repair, which is the standard treatment. If immediate resources for repair are unavailable, the procedure can be delayed for up to 12 h without significant adverse effects [33]. Extensive tears and all third- and fourth-degree lacerations must be repaired under general or regional anesthesia, ideally in the operating room. Muscle relaxation is necessary to align and overlap the retracted muscle ends without tension. Prophylactic antibiotics should be administered. The torn anal epithelium is repaired with interrupted 2-0 polyglactin sutures, with knots tied inside the anal lumen. The ends of the external anal sphincter (EAS) are fully mobilized and repaired using the overlap technique, with 2-0 polydioxanone being the preferred suture material. Since no clear evidence supports one method over the other, surgeons may opt for either an end-to-end or overlap repair of the EAS [18]. Whenever possible, the internal anal sphincter should be repaired separately using interrupted 2-0 polydioxanone sutures, with either the end-to-end or overlap technique.

If the rectal mucosa is torn, it should be repaired using 2-0 polyglactin for interrupted sutures or 2-0 polydioxanone for continuous submucosal sutures. The perineal muscles and subcutaneous tissue are repaired with 2-0 polyglactin, while the perineal skin is closed using a subcuticular or interrupted polyglactin suture. A rectal examination must be conducted at the end of the procedure to confirm the integrity of the repair, and the results should be documented. The rates of wound infection and dehiscence after primary OASI repair are 4% and 7%, respectively [34]. Given the risk of wound complications following primary OASI repair, the Royal College of Obstetricians and Gynaecologists (RCOG) recommends prophylactic broad-spectrum antibiotics. However, the American College of Obstetricians and Gynecologists does not support this practice due to the lack of high-quality research demonstrating postpartum clinical benefits from antibiotic use.

On rare occasions, a temporary stoma could be created to allow the compartments to be cleaned and the laceration reconstructed. Once healing has occurred, the stoma can be removed [18].

### 3.3. Fertility and Pregnancy in Patients with IBD

#### 3.3.1. Incidence

The incidence of inflammatory bowel disease (IBD) in Western countries is approximately 0.7%, with the peak age of diagnosis occurring during the reproductive period [35,36,37]. Around 50% of patients are diagnosed with IBD before the age of 35; fertility is a major concern for these patients [38], as an increasing number of women plan a pregnancy after being diagnosed with IBD. Pregnancy often correlates with stress and anxiety in healthy women; in women with IBD, stress related to pregnancy outcome leads to a significantly higher rate of voluntary childlessness [39]. Women make this choice mostly due to a lack of factual and thorough information regarding pregnancy and IBD, indicating the need for pre-conception counseling and support during pregnancy and delivery [40].

#### 3.3.2. Preconception

The real impact of IBD and its specific therapies on fertility and pregnancy is not known by most women, and the real effect of a pregnancy on the severity and management of IBD must be clearly explained. Preconception counselling should always be provided to explain not only the correct control of the disease during conception and gestation but also the most appropriate nutritional and healthy behaviors during this period [41,42]. Indeed, a dietary specialist should adjust the dietary guidelines of pregnant women with IBD to avoid inadequate caloric intake, which therefore increases the risk of low fetal intrauterine growth during gestation [43]. Very insufficient clinical data is available for pregnant and/or breastfeeding women with IBD, due to their exclusion from clinical trials, and the few published results originate from cohort studies and case series that can hardly generate clinical guidance [44]. Several studies evaluated the implication of IBD on the outcome of pregnancy, fertility, and the perinatal outcomes of the newborn. However, the results have been heterogeneous due to the variability of this condition [38].

#### 3.3.3. Fertility

Infertility is defined as the inability to conceive after 12 months of unprotected intercourse. According to the latest guidelines from the European Crohn’s and Colitis Organization (ECCO) on reproduction, ulcerative colitis (UC) without a history of pelvic surgery and inactive Crohn’s disease (CD) do not negatively affect fertility [45]. However, an active disease or a long history of disease has a marked impact on the reproductive system [46,47].

Surgery adversely affects fertility; colectomy with end ileostomy had the least impact, followed by IRA, while a stronger correlation between ileal-pouch–anal anastomosis (IPAA) and an increased risk of infertility has been reported [48,49]. IPAA surgery may impair fertility due to adhesions, thus increasing tubal dysfunction. Infertility rates rise from 15–20% pre-IPAA to 48–63% post-IPAA [50]. This risk applies to females only since a recent study found no difference in male fertility: sperm count, motility, vitality, or morphology in men with IBD are comparable to the general population after stopping sulfadiazine for at least 3 months [51,52]. However, it is noteworthy that pregnancy rates are significantly higher after laparoscopic IPAA when compared to open surgery, possibly due to reduction in adhesions to the tuboovarian complex. For this reason, the laparoscopic approach should be considered the method of choice in young women [53].

Fertility does not appear to be affected in women with inactive IBD as a result of in-vitro fertilization [46]. Due to the potential negative outcomes correlated with active disease and fertility, patients with IBD may consider an earlier use of technologies for assisted conception compared to general indication, even after only six months of attempts [54].

#### 3.3.4. Pregnancy

Pregnant patients with IBD tend to experience improved modulation of cytokine patterns, with inflammation marker levels gradually decreasing throughout gestation. However, babies born to mothers with IBD may have a reduced capacity to develop balanced mucosal immunity or establish optimal intestinal barrier function, potentially increasing their risk of developing IBD later in life.

Actually, there is no evidence that pregnancy is correlated with disease progression. European studies showed that pregnancy is not only associated with a better course of disease but also with a reduction in flares over time and with a reduction in surgical resections [55,56,57]. An interesting correlation exists between IBD activity at the time of conception and during gestation. Two-thirds of patients with inactive IBD at the time of conception remain quiescent during the entire pregnancy, and the risk of relapse is 20–30%, like in non-pregnant patients [58]. In IBD patients, immunological parameters improve during pregnancy, and microbial diversity returns to levels similar to those seen in healthy pregnant women. However, children born to mothers with IBD display an altered gut microbiome, which is associated with abnormal adaptive immune systems and an increased risk of developing IBD in the future [59]. Therapeutic management in these patients is in constant evolution. As adequate control of the disease is essential during pregnancy, medications and monitoring during antenatal, pregnancy and postpartum stage should be personalized for each patient’s need and history [40]. Therefore, close monitoring of inflammatory markers and fecal calprotectin should be undertaken. When needed, endoscopy and imaging [ultrasound and MRI) should also be performed [60,61]. Endoscopy without oral bowel preparation can be safe in pregnancy if it is correctly indicated [62]. Medical management of IBD is essential to optimize outcome for both pregnant woman and the fetus. Most of the state-of-the-art therapies are safe to be continued during gestation, although many women decide to interrupt them, before or during pregnancy, for fear of fetal or pregnancy consequences [63]. The safety of 5-ASA compounds was studied in several trials. Women taking 5-ASA, as well as mesalamine and sulfasalazine, for IBD, do not show higher incidence of fetal abnormalities compared to the general population since these drugs have been demonstrated to be safe in pregnancy [64]. Current guidelines advise maintaining anti TNF-α treatment during pregnancy for both women with active disease and those in remission [65]

As far as biological drugs that change the course of IBD, increasing the number of remissions, they can be safely used in pregnant women. A recent metanalysis suggested that infliximab (IFX), adalimumab (ADA), and other new non-TNF biologics such as vedolizumab and the monoclonal antibody ustekinumab (UST) have little or no impact on pregnancy or fetus. Although many biologics cross the placenta and can be found in fetal circulation, their presence does not affect fetal development, nor does it increase the risk of fetal malformation, low birth weight, or the risk of stillbirth, preterm birth, or early pregnancy loss [66]. IFX and ADA, two IgG1 monoclonal antibodies, can cross the placenta and be found in breast milk and infants after birth, without any reported adverse effects. However, more research is needed on UST, the anti-IL-12/23 agent, before clear recommendations can be made [67]. Overall, the data reported support safe use of biologic drugs during pregnancy, if required to control IBD activity, [68] recognizing that in the uncontrolled disease, there is the presence of worse pregnancy and fetal outcomes. Finally, it should always be emphasized that all the studies in the literature present some bias due to the lack of randomized studies and the heterogeneity of the results.

As far as pregnancy outcome is concerned, a former study in 1980 by Willoughby et al. reported how patients with inactive IBD do not have an increased risk of adverse pregnancy outcome compared to mothers with active disease [69]. On the other hand, subsequent studies reported that the perinatal risk could be as high as 35% in mothers with active IBD, including low birth weight, spontaneous abortion, preterm delivery, stillbirths [70,71], and other perinatal outcomes such as intra-amniotic infections, cytokine-mediated premature labor, and abnormal development of the fetus [72].

#### 3.3.5. Delivery

There are no guidelines in the literature on the best method of delivery; however, C-section tends to have a higher frequency in women with IBD compared to healthy woman [49] (25% vs. 9.5–28.2%) [73,74].

Vaginal delivery is safe for women with inactive IBD; actually, in this subset of patients, the mode of delivery should be guided only by gynecological and obstetric criteria. Conversely, women with active disease have a more frequent operative delivery. In women with perianal disease or previous surgery (especially in case of IPAA) or with a long-term illness (diagnosed over 5 years before), there is a higher risk of injuries due to the increase in sphincter pressure [75,76]; therefore, they should be directed to C-section delivery, in agreement with ECCO guidelines [77]. Accordingly, in patients with previous surgery or presence of a stoma (colostomy or ileostomy), the mode of delivery should be considered with a multidisciplinary consultation. Key factors to be considered in the decision are the increased risk of intestinal obstructions and associated possible obstetric complications during labor due both to the presence of the stoma and to the possible presence of peritoneal adhesions [78].

### 3.4. Pregnancy, Delivery, and Hemorrhoid Disease

#### 3.4.1. Prevalence

Pregnancy and the postpartum period can be significantly affected by anal symptoms, often severe, due to hemorrhoidal disease (HD). The prevalence of HD during pregnancy ranges from 24% to 35% [79,80,81,82], with estimates reaching up to 85% [83], particularly during the second and third trimesters. HD in pregnancy may show different presenting symptoms. The most frequent presentation is marginal thrombosis, whereas prolapse, which can lead to obstructed defecation, is less frequent.

In a prospective observational study by Poskus et al., 40.7% of women were diagnosed with hemorrhoids, and 2.5% had both hemorrhoids and anal fissures during pregnancy and after delivery. The prevalence of perianal disease was 1.6% in the first trimester, 61% in the third trimester, 34.1% post-delivery, and 3.3% one month after delivery, with hemorrhoids being the most common condition [84].

#### 3.4.2. Risk Factors

Several factors have been proposed to explain the development of hemorrhoidal disease (HD) during pregnancy, including increased pelvic pressure, obstruction of pelvic venous outflow, the relaxing effect of progesterone on venous walls, increased circulating blood volume, and constipation [85,86]. Additional predisposing factors include age and parity [81,82]. In a recent study by Bužinskiene D. et al., further risk factors were identified, such as prolonged pregnancy (over 40 weeks), prolonged labor or straining for more than 20 min, instrumental delivery, and newborn weight over 3800 g [87]. Despite the different etiological factors in pregnancy, the guidelines for preventing and treating hemorrhoids are the same as those for the general population, with a primary focus on bowel management. This includes dietary changes and fluid and fiber intake [88], though the results are sometimes inconsistent [89]. Other recommended treatments include sitz baths or topical ointments [90,91], while the use of flavonoids is not supported due to insufficient evidence of their safety during pregnancy [82,90,91,92,93].

While childbirth often initiates the spontaneous resolution of HD [94], the postpartum period sees the highest rate of thrombotic hemorrhoidal events, leading to a worsening of symptoms (7.8% in the third trimester compared to 20% post-labor) [95]. In a randomized trial, Poskus et al. demonstrated that hemorrhoids before pregnancy and the increase in newborn height were associated with a higher risk of hemorrhoids after delivery; nevertheless, specific dietary and behavioral counseling during pregnancy significantly reduced the rate of hemorrhoids at the time of discharge from the obstetrics unit [96]. Åhlund et al. interviewed a group of women 3 weeks and 1.5 years after the first delivery; half of the women who reported problems with hemorrhoids 3 weeks after birth still experienced problems after 1.5 years. Although hemorrhoids were perceived as a problem that affected their quality of life, they felt that this problem was neglected by the healthcare system and suggested that more information should be given from the midwives during pregnancy, after birth, and at the postpartum check-up [97].

#### 3.4.3. Treatment

Since HD occurs in approximately 50% of pregnancies, a proctological screening should be scheduled in the second and third trimesters, particularly for patients with a prior history of HD. Additionally, there may be a correlation between the development of HD and other postpartum perineal issues, such as stress urinary incontinence or pelvic organ prolapse [98,99], which should also be investigated.

Despite the standardized approach to HD in the general population, as outlined by various scientific society guidelines [95,100,101,102], symptom relief during pregnancy relies on a limited set of empirical recommendations related to bowel habits, diet, topical ointments, and fluid intake. Currently, there are no specific therapeutic measures available for treating HD in pregnancy, and treatment options are based primarily on conservative and empiric approaches. Further research is needed to identify targeted and effective treatments for this condition.

In a recent multicenter study [102], pregnant women were advised with a whole package of recommendations (diet, fluid intake, exercise, etc.). Such counseling significantly reduced the rate of HD in pregnancy. However, although these simple methods may provide some degree of symptom relief, they do not address the underlying cause of the condition. Surgery could be an option in very selective cases. Saleeby [98] performed hemorrhoidectomy on pregnant women, and in 24 out of 25 patients, significant symptom relief was achieved following the removal of thrombosed or gangrenous hemorrhoids. Therefore, considering the limited effectiveness of conservative empirical methods, it is not surprising that hemorrhoidectomy during pregnancy appears to be a highly effective treatment for select patients with hemorrhoid complications; in these circumstances, hemorrhoidectomy could also be performed safely under local anesthesia [103]. Prolapse and ODS are amenable to dedicated treatment after delivery, if persistently symptomatic.

### 3.5. Urinary and Sexual Dysfunctions after Rectal Cancer Surgery

#### 3.5.1. Introduction

When curative surgery for rectal cancer was introduced 100 years ago, radical abdominoperineal resection (APR) was invariably associated with urogenital dysfunctions because of its mutilating nature [104]. By the 1940s, it was understood that genitourinary function depends on the integrity of the pelvic autonomic nerve plexuses [105]. The last few decades have seen significant improvements in the treatment of rectal cancer with a shift from amputation surgery to sphincter saving procedures, which led to decreased functional postoperative complaints and improved quality of life. With improved prognosis and with the growing number of survivors, treatment-related morbidity has become increasingly significant. It is well established that multimodal treatment for rectal cancer can disrupt normal pelvic organ function, leading to a reduced quality of life [106]. However, there is little information and few well-conducted studies on the urinary and sexual dysfunctions of patients treated for rectal cancer, and, in addition, studies performed to date have focused mainly on urogenital function in men [107,108]. Moreover, many studies involve patients who received neoadjuvant therapy, making it challenging to isolate the effects of surgery alone on female sexual and urinary function.

It is also more complex to identify a suitable criterion for measuring changes in sexual activity over time in women than identifying the evaluation criteria in men, whose main targets are erectile function and retrograde ejaculation. In most of the studies, female sexual function is simply related to sexual activity [109,110,111], dyspareunia [109], loss of libido [112], or reduced ability to achieve orgasm [110,113] after rectal cancer excision. Urinary dysfunctions after rectal cancer treatment are represented by urge incontinence (detrusor instability or reduced bladder capacity), overflow or stress incontinence (reduced support of the urethra and bladder neck) and retention, as well as minor complaints such as difficulty initiating urination, sensation of incomplete urination, pain and discomfort during urination, increased urinary frequency, and/or nicturia.

#### 3.5.2. Anatomy-Pathophysiology

Pelvic autonomic nerve lesions underly sexual and urinary dysfunctions. The superior hypogastric plexus is located 3–7 cm below the origin of the inferior mesenteric artery, adjoining the aortic carrefour; the thin fibers of the plexus divide into two nerves, the right and left hypogastric, running medially to the ureters to connect to the inferior hypogastric plexus. The inferior hypogastric plexus appears as a network triangle, anterolateral to the rectum and lateral to the cervix, vaginal fornix, and bladder, often extending into the broad ligaments of the uterus; in addition, the inferior hypogastric plexus has connections with sacral nerve roots from S2 to S4 through the parasympathetic pelvic splanchnic nerves. The nerves follow the inferior hypogastric plexus to the bladder and sexual organs via neurovascular bundles located along the lateral edge of Denonvilliers’ fascia, near the anterior rectal wall [114]. Besides nerve damage, postoperative scarring around the vagina may lead to discomfort or avoidance of sexual intercourse. Low rectal dissection requires full mobilization of the recto-vaginal septum, and studies have shown that women who underwent pelvic surgery often reported feeling that their vagina was inelastic or too short during intercourse [109,115]. These issues can be further aggravated when partial excision of the vaginal wall is required for oncologic purposes. Advanced sphincter-sparing procedures, such as intersphincteric dissection or coloanal anastomosis, may worsen sexual function by causing fibrosis and perineal distortion, potentially reducing vaginal compliance and resulting in painful intercourse. Additionally, abdominoperineal resection has been linked to changes in bladder position within the pelvis and muscle pain during intercourse.

#### 3.5.3. Epidemiology

A literature review on the impact of aging on sexual function concluded that older women treated for rectal cancer experience a decline in sexual activity and desire and that this decline usually begins in the fourth decade [116]. Although about 1/3 of women aged 50–70 years report lack of sexual desire, in women treated for rectal cancer, the lack of sexual desire is present in 60% of patients. Furthermore, QoL improves with survival duration [117]. The rate of urinary dysfunction, on the other hand, after rectal cancer surgery, currently ranges from 30% to 70% of treated women [118,119]. Diriment is the Dutch study on TME [120], which detected, in the 5-year follow-up, urinary incontinence and bladder emptying difficulty in 53.2% and 26.5% of patients, respectively.

#### 3.5.4. Risk Factors

Factors predisposing to postoperative urinary and sexual dysfunctions include age, duration of surgery, use of high doses of opioids for analgesia [121], tumor location (particularly of the lower third) [122], preexisting urinary disorders, excessive intraoperative blood loss [123], and fibrosis caused by radiotherapy [124,125,126]. Several studies have demonstrated an incremental increase in urinary dysfunctions in women after radiation therapy for rectal malignancies compared with women who undergo upfront surgery. Radiation therapy may have a detrimental effect on female sexual function as well, with increased incidence of dyspareunia, loss of libido, and fatigue; all these symptoms are most likely caused by diffuse fibrosis, mucosal irritation, and premature pre-menopause induced in younger patients; this may result in premature ovarian failure, with the loss of the ability to achieve orgasm and postcoital bleeding [127,128].

Long-term consequences of postoperative intra-abdominal sepsis have been shown to significantly reduce the ability to achieve arousal and orgasm, compared to patients who experience an uncomplicated postoperative recovery [129].

#### 3.5.5. Intervention (Type and Technique)

Before the introduction of total mesorectal excision (TME), the incidence of sexual and urinary dysfunctions was high, 10–30% and 40–60%, respectively [130,131]. TME involves precise dissection under direct visualization along pre-existing embryological planes, separating the visceral fascia surrounding the mesorectum from the pelvic parietal fascia that covers the pelvic floor [132]; the TME technique led to reduced local recurrence rates (>20% to <10%) and improved survival (48% to >60%). Women undergoing TME, however, may experience damage to the hypogastric plexus, resulting in a loss of sympathetic innervation, which can lead to urgency and stress urinary incontinence. The modification proposed by Hojo and Moriya, in which the dissection is medial to the autonomic nerves and not only peripheral to the mesorectum, preserves the autonomic nervous system and reduces the incidence of sexual and urinary complications, 10–35% and <5%, respectively [110,133,134]. Few studies have specifically examined the impact of surgical treatment for rectal cancer on pelvic dysfunction in women. Daniels et al. followed up with women after TME and found that 59% experienced nocturia and 18% had stress incontinence post-surgery. These symptoms were more prevalent in women with low rectal cancers [135]. No difference was found, however, with respect to urinary dysfunction and its impact on quality of life between women undergoing partial mesorectal resection and those treated by TME [136]. With the advent of laparoscopic surgery, it was anticipated that the enhanced, magnified view would aid in preserving nerves, thereby improving urinary and sexual function. However, several studies have shown that the rate of dysfunction in the laparoscopic TME group is comparable to, or even higher than, that of the open surgery group [137,138]. Robotic TME offers advantages over laparoscopic surgery, including a magnified view, greater dexterity with wristed instruments, and improved ergonomic comfort. These features could potentially enhance the identification and preservation of autonomic nerves compared to conventional open and laparoscopic surgery. However, there is currently no evidence that robotic surgery is superior to conventional laparoscopy in performing nerve-sparing TME for rectal cancer [139].

As far as the comparison between anterior rectal resection (AR) and abdominoperineal resection (APR), it should be noted that APR involves partial resection of the levator muscles of the anus and increases the risk of surgical damage to pelvic autonomic nerves [110,129,130,140] in addition to a change in the position of the bladder in the pelvis [141]. In a prospective study of 295 women who underwent rectal cancer surgery [142], less than half were sexually active one year post-surgery. Among those who were, a significantly higher proportion had undergone anterior resection (AR) compared to APR. APR was linked to decreased sexual activity, a higher incidence of dyspareunia, and poorer urinary function, including symptoms of urgency, incontinence, altered flow, and retention episodes.

Another important aspect concerns pelvic lymph node dissection associated with rectal resection surgery that adversely affects urinary function, the severity of which is determined by the extent of such dissection. Mechanical damage to nerve fibers and ischemic damage caused by devascularization during dissection are also believed to play an important role [143,144].

#### 3.5.6. Management

The management of patients with urogenital dysfunction after rectal cancer treatment is still poorly supported. Primarily, pelvic floor muscle training has been shown to be useful for patients with intact pelvic floor innervation. When conservative management fails, both sacral nerve stimulation [144] and the interposition of a nerve graft could be employed with good results; the nerve graft operates mainly as a scaffold for regenerating axons to reestablish the connection between severed segments [145]; this technique is, however, still experimental.

There is a lack of research on the effects of rectal cancer and its treatment on urinary dysfunction, sexual activity, and overall quality of life (QoL) in women. Often, information has been gathered using unvalidated questionnaires, which limits reliability and misses important aspects like sexual desire, lubrication, and overall satisfaction. A Danish study addressed this by developing and validating a simple scoring system to assess sexual function in women treated for rectal cancer. This system effectively captures the key issues of female sexuality from the perspective of rectal cancer survivors with high sensitivity [146].

### 3.6. Rectovaginal Fistula

Rectovaginal fistula (RVF) is a pathological epithelium-lined communication between the anal canal or the rectum and the vagina; it is responsible for uncontrolled passage of gas and purulent or fecal discharge from the rectum to the vagina, with consequent recurrent vaginal or lower urinary tract infections. Symptom severity is related to the site and the size of the fistula: small anovaginal fistulas may cause few symptoms, whereas larger and higher fistulas can result in severe distressing symptoms that have a dramatic impact on the physical, psychological, sexual, and social life of the affected women, especially in case of RVF due to Crohn’s disease, radiotherapy, or cancer. The low success rate and the frequency of recurrences make treatment of RVF challenging for surgeons and an intolerable condition for patients.

#### 3.6.1. Etiology

The most common cause of RVF is obstetrical trauma (88%). Third- and fourth-degree lacerations, along with median episiotomies, are known causes of fistula formation [147] in 1% of cases after repair [148]; higher rates are registered in developing countries [149]. Crohn’s disease is the second common cause of RVF formation, with an incidence of 9–10% in affected women [150,151]; RVF can occasionally occur in ulcerative colitis and complicated diverticular diseases. Other causes include local infections (cryptoglandular and Bartholin gland abscesses); irradiation of the pelvis (particularly for gynecological malignancies); local extension of anorectal, cervical, vaginal, or vulvar neoplasms; postsurgical complications following pouch construction; low anterior rectal resection; rectocele repair; and hysterectomy. An increasing incidence has been reported in the last decades due to the diffusion of stapled hemorrhoidopexy and STARR procedures [152,153,154].

#### 3.6.2. Classification

RVF is generally classified according to location of the vaginal opening (low: near the posterior vaginal fourchette; middle: between the uterine cervix and the posterior fourchette; high: in the posterior fornix), size (small diameter: <0.5 cm; medium: 0.5 to 2.5 cm; large: >2.5 cm), and etiology. In surgical practice, an RVF is classified as “complex” if it is large, high, caused by IBD or other pelvic processes or irradiation, and recurrent. Conversely, a “simple” RVF has a small diameter, is located distally from the anal sphincter complex, and is mainly due to obstetric injury or cryptoglandular infection. Some authors have classified low RVF as an anovaginal fistula if the opening is located at the vaginal introitus and arises from the anal canal [155].

#### 3.6.3. Evaluation

Symptoms of RVF may vary depending on size, location, and etiology, as well as stool consistency and patients’ tolerance. Clinical assessment with digital rectal and vaginal examination and anoscopy and speculum examination is crucial to identify a low or middle RVF. In addition, methylene blue rectal retention enema, administered with a tampon placed in the vagina, may be useful to demonstrate an unidentified communication. Examination under anesthesia may be necessary in case of suspected malignancy or in patients with radiation injury. Endoanal ultrasound, with the addition of hydrogen peroxide as a contrast medium, is useful to identify the fistula tract, assessing the width of the rectovaginal septum and the integrity of the anal sphincters, which play an important role in the choice of surgical repair [156]. The examination of the lesser pelvis by CT scan or MRI has a pivotal role in identifying associated pathological conditions, especially malignancies [157]. Other radiological evaluations, such as contrast vaginography, fistulography and barium enema, or endoscopic procedures, may be of value but are currently performed with reduced frequency. Manometric evaluation of the anal sphincters is mandatory to choose the best surgical option.

#### 3.6.4. Treatment

The initial management of RVF is conservative; it is based on baths, wound care, debridement in case of infection (eventually with draining seton placement), antibiotics if required, and stool-bulking fiber supplementation to reduce the passage of liquid stool in the vagina, for a period of 3–6 months. In patients with RVF due to obstetrical trauma or benign and minimally symptomatic fistulas, the healing rate with a conservative approach ranges from 52% to 66% [158,159].

A large variety of surgical techniques has been described for RVF repair through different approaches: transanal, transvaginal, transperineal, and abdominal routes. However, high recurrence rates are reported for each treatment. Most published studies are limited by the small sample size and the heterogeneity of etiology and surgical treatment techniques. No randomized trials are available on surgical treatment of RVF [160]. The only available guidelines are the German Guidelines [161] and the American Society of Colon and Rectal Surgeons clinical practice guidelines [158], which are based on low-quality evidence.

The choice of treatment should be driven by size, location, and etiology of RVF, as well as the status of the surrounding tissue and the anal sphincter complex integrity. Previous failed repairs and surgeon’s experience are also important factors to take into account. The surgical approach must strictly follow key principles, including wide dissection around the fistula, mobilization of surrounding tissue, removal of scar tissue, complete excision of the fistula tract, and ensuring a tension-free repair.

#### 3.6.5. Endorectal Repair

The procedure of choice for most simple RVFs is advancement flap with or without sphincteroplasty. A wide-based flap of rectal mucosa, along with a small amount of underlying sphincter muscle, is mobilized and advanced to cover the defect in the rectovaginal septum. Lack of tension and good perfusion are crucial for flap success, which ranges from 41% to 78%. Causes of failure include undetected sphincter defect, Crohn’s disease, radiotherapy, and recurrent RVF. Even in the case of recurrence, success has been reported by repeating the advancement flap. Adding sphincteroplasty to the procedure increases the success rate [162]. An alternative approach to treat low RVF is the advancement of a flap harvested from the anoderm and perianal skin. As for the treatment of anal stenosis, several flap configurations have been described. The anodermal advancement flap appears to allow better healing, good blood supply, and less retraction than a mucosal flap, even if this is currently not supported by literature [163].

#### 3.6.6. Vaginal Repair

After primary closure or inversion of the fistula into the rectum by means of a purse-string suture, a large flap of vaginal mucosa is mobilized and used to cover the fistulous orifice. Gynecologists report acceptable healing rates with this procedure. However, these are questionable, considering that the higher intrarectal pressure creates a gradient that pushes feces from the rectum to the vagina, thus compromising healing and facilitating recurrences. The positive results reported could be explained by the fact that most patients already had a diverting stoma at the time of surgery [164]. Very few publications are available, and no recommendation can be made based on current literature.

#### 3.6.7. Transperineal Repair

Episio-proctotomy is a good surgical option with acceptable functional outcome in patients with obstetrical or cryptoglandular RVF associated with extensive anal sphincter defect. The tissue above the fistula (perineal skin, external anal sphincter, and rectovaginal septum) is cut in full thickness, converting RVF in a fourth-degree laceration. The tissue is then reconstructed in layers after fistulotomy. An overlapping sphincteroplasty is performed [165]. The healing rates vary from 35% to 100% in a small series of patients [158].

#### 3.6.8. Tissue Interposition

The interposition of healthy, well-perfused tissue between the rectal and vaginal walls, following direct closure of the corresponding fistula orifices, is indicated in the case of repeated failures of RVF repair, or when tissues are severely damaged or replaced by scars to begin with. The interposed tissue acts by separating the vaginal suture from the rectal suture. The Martius procedure employs an adipose tissue pedicled flap mobilized from the labia majora. The interposition of bulbocavernosus muscle in order to increase the thickness of the traditional Martius fat flap has been reported in some studies, with a success rate that ranges from 65% to 100% [166]. The advantage of this procedure is the lower morbidity in comparison to other tissue interpositions, despite frequent postoperative dyspareunia.

Gracilis muscle flap interposition is more invasive and complex than Martius bulbocavernosus flap; for this reason, it should be considered as a second- or third-line treatment for patients at low risk of recurrence. In patients at high risk of treatment failure, this procedure could be considered as a first-line therapy. A recent systematic review [167] reports a success rate of 64% (range 33–100%); risk factors for failure identified in various studies are smoking, Crohn’s disease, and more than two previous failed repairs. Many authors believe that a diverting stoma is an important factor for gracilis muscle interposition success.

#### 3.6.9. Abdominal Repair

The transabdominal approach is reserved for higher RVFs, usually resulting from colorectal anastomotic complications. Different surgical options are available, such as redo colorectal anastomosis with endorectal advancement flap or transperineal interposition flap, or fecal diversion alone [168]. Proctectomy with or without pull-through or coloanal anastomosis is indicated in RVFs that are radiation-related or in complex multi-recurrent fistulas. Diverting stoma without proctectomy can relieve symptoms, but the association of the two procedures significantly improves quality of life, causing reduction in tenesmus and anal discharge [169].

#### 3.6.10. Other Treatments

Endoscopic repairs, including stenting, clipping, and transanal endoscopic microsurgery, have been recently reviewed, with a success rate ranged from 40% to 93% for middle and low fistulas [170]. Treatment results using fibrin glue, fistula plug, and stem cells therapy, mainly proposed for Crohn’s RVFs, are usually disappointing [161,171].

#### 3.6.11. Stoma

The need for fecal diversion in the setting of RVF treatment remains a source of debate; nonetheless, it is generally accepted that diverting stoma can reduce the impact of symptoms and might help with fistula healing. The clinical effectiveness of diverting stoma remains controversial due to insufficient proof from literature. In general, the decision regarding stoma creation should be based on the extent of damaged tissue and the severity of local inflammation, the amount of fistula discharge, and the resulting burden on the patient [172].

## 4. Discussion

When faced with coloproctological disease in women, one should consider three different aspects. The first aspect is related to specific problems due to anatomical or physiological differences between men and women. In this group, we considered rectovaginal fistula, endometriosis, and OASIs. RVF is a challenging clinical problem that dramatically affects a patient’s quality of life [172]; this condition is difficult to manage, with a poor level of evidence for surgical approaches; the evidence is mainly based on case series with no RCT and only two societies’ guidelines [158,161] based on low-quality evidence; therefore, no specific procedure can be recommended based on the literature. Accordingly, high-quality studies should be implemented to better address the approach to this challenging condition. Endometriosis is an even worse condition, often requiring complex surgical procedures and specific expertise [173]. Although the QoL significantly improves after surgery [15], surgical treatment is still hampered by functional consequences with the persistence or new development of trubelsome symptoms. Safer alternative options that could provide more timely care to women should be the focus of further studies [174]. The final condition, OASIs, still affects 3% to 5% of all vaginal deliveries, possibly leading to significant comorbidities, including rectovaginal fistula, urinary retention, and pain, and is a leading risk factor for subsequent loss of bowel control in women. Guidelines exist to improve diagnosis, pursue optimal functional outcomes following repair, and provide evidence-based counsel to women for future childbirth; these guidelines should be implemented to decrease the occurrence or improve the functional consequences of these lesions.

The second aspect pertains to problems common to men but poorly studied in women. All the urinary and sexual dysfunctions after rectal resection belong to this group. The majority of information on this topic derives from studies performed on men, and information on function and quality of life in women is lacking; additionally, urinary function and sexual activity in women have been studied with unvalidated questionnaires, thus impairing the reliability of the results. Accordingly, no treatment protocols have been developed. The diffusion of a simple and validated scoring system [146] to assess sexual function, recently developed, could help to better understand and possibly treat these dysfunctions.

Finally, there is a third group of diseases, common to men, whose etiology and treatment are poorly studied when occurring during pregnancy. Anal symptoms, mainly related to hemorrhoids, occurring in pregnancy and postpartum, although self-limiting in the majority of cases, could be severe enough to affect the quality of life of affected women; the classic risk factors observed in the general population seem not to be applicable to pregnant women, and therefore specific therapeutic measures are not usually considered. No guidelines suggest a standardized approach to HD in pregnancy; therefore, studies are needed in order to identify a targeted etiologic treatment.

Another demanding and neglected issue is the management of pregnancy in women with IBD. It seems that a bidirectional interaction strictly correlates IBD and pregnancy: IBD affects fertility and pregnancy outcome, while pregnancy has implications for IBD activity. Managing pregnancy in women with IBD can be complex due to limited data and concerns regarding medication toxicity; improper management may increase the risk of disease flares and adverse outcomes for these women; however, fertility and pregnancy seem not particularly affected in women with IBD; during pregnancy, biological drugs can safely be continued since they have little impact on fetus [65,66]; actually, mismanagement can put women at risk of disease flares and adverse outcomes since women with active disease have an increased risk of preterm delivery, babies with low body weight, and cesarean section delivery. As far as the mode of delivery is concerned, there are no guidelines in the literature on the best way of delivery in women with inactive disease. ECCO guidelines [77] provide suggestions for a C-section delivery only for patients with previous surgery or perianal disease due to the higher risk of complications. A multidiscilplinary consensus is warranted in these circumstances [78].

Hence, it emerges from the literature that more high-quality studies are needed in order to draw up guidelines and treatment protocols to provide effective care tailored to women’s specific health experiences.

Every contribution to gender medicine is crucial, both in Italy and globally, for several reasons: by addressing health disparities, it may promote research and practice that considers gender differences, thus leading to more effective treatments; it can promote awareness for clinicians, leading to more personalized treatments; it can also inform healthcare policies, ensuring that they are more inclusive and responsive to the needs of all genders.

The present article, by highlighting several gender-specific issues in the field of coloproctology, could contribute to the development of gender medicine, as it underlines specific risk factors, enhances a more comprehensive understanding of women’s health, and informs and guides healthcare providers in recognizing and addressing gender-specific symptoms. The article can also serve to identify research gaps and to encourage ongoing investigations in the field.

Our paper has some limitations: first, the choice of topics was based primarily on experts’ opinions, and some conditions may have been overlooked. Secondly, the conditions addressed are very different from one another, and for this reason, the text may seem fragmented. Finally, the research was based on a single database (PuBMed), thus being less likely to cover all the relevant literature. Nevertheless, this is the first article addressing gender differences in coloproctological diseases, not only being crucial for healthcare professionals, as it highlights the need for personalized treatment approaches, but also responding to precise national government directives as well as recommendations by international organizations, such as the World Health Organization, the European Community, and the European Medicine Agency, providing information for public health policies and resource allocation, and supporting the development of gender-sensitive healthcare initiatives.

This can lead to more effective care, better patient outcomes, and a deeper understanding of disease mechanisms.

## 5. Conclusions

In conclusion, this review described the presentation, diagnosis, and management of common coloproctological disorders, focusing on women.

The article highlights the need for personalized treatment approaches based on gender-specific factors. This can lead to more effective care, better patient outcomes, and a deeper understanding of disease mechanisms. For governments, such research can inform public health policies, allow resource allocation, and support the development of gender-sensitive healthcare initiatives, ultimately improving population health equity.
